# Dispatch centres: what is the right population catchment size?

**DOI:** 10.1186/s13049-015-0111-5

**Published:** 2015-04-09

**Authors:** Fabrice Dami, Vincent Fuchs, Olivier Hugli

**Affiliations:** Emergency Medical Services, State of Vaud (Fondation Urgences-Santé), Lausanne, Switzerland; Department of Emergency Medicine, University Hospital Centre (CHUV), Lausanne, Switzerland

## Abstract

Literature on medical dispatch is growing, focusing mainly on efficiency (under and overtriage) and dispatch-assisted CPR. But the issue of population catchment size, functional costs and rationalization is rarely addressed. If we can observe a trend toward a decreasing number of dispatch centres in many European countries, there is today no evidence on what is the right catchment size to reach the best balance between quality of services and costs.

## Discussion

During the last 15 years, an increasing number of articles on medical dispatch has been published. Today, some journals are even dedicated only to this link of the chain of survival [1,2]. The main topics treated focus primarily on protocols and dispatcher effectiveness (under- and over triage) and cardiac arrest (recognition and dispatch-assisted CPR). Surprisingly, the issue of catchment population size is rarely addressed.

Dispatch centres (DCs) are considered as an essential but expensive component of many highly developed health-care systems. The number of DCs in a country, a region or a state, are usually based on local history and often related to highly decentralized health-care systems. Most communities have built their own dispatch at a time when no such service existed. Today, current technology (global positioning system or Internet access) abolishes the need for closeness between DCs and the population. It is theoretically and technically possible to serve a state/region from anywhere in the world. Foreign languages seem to be the only rational barrier to the globalization of this activity. However, most communities are reluctant to abandon their DCs, just as it is sometimes difficult to merge police forces or fire services from different states/regions, for non-rational reasons.

For example, Switzerland is a country of 41,285 km2 (15,940 square miles). It is among the richest countries in the world [3] and has a system of public health surpassed only by the USA in cost per capita [4]. As in the USA [5] or in Germany [6], the health-care system is fragmented and highly decentralized. Each of the 26 states of the Swiss federation is sovereign regarding its health-care system, including its prehospital system [7]. In 2006, there were 22 DCs for a population of 7.5 million. Each DC served on average 340,000 inhabitants. As Swiss regulation requires a minimum of two dispatchers per DC at all times, that means there were 44 dispatchers working every night in the country. As with anywhere else, there is currently pressure on costs in all health-care sectors, including prehospital systems where medical dispatch centres are expensive resources to run. This is the main reason Switzerland went from 22 DCs in 2006 to 17 today serving 8 million inhabitants.

Although evidence is lacking regarding the most appropriate catchment population per DC, several European countries have also been decreasing their total number of DCs (Table 1). Table 1 also shows examples of population catchment of some major cities’ DCs worldwide, some reaching more than 4 million inhabitants.

**Table 1:** **Medical dispatch centres**

	**Year**	**Population in millions**	**Number of medical dispatch centres**	**Average population per dispatch in million**
**Countries:**				
Iceland (1)	2004	0.3	2	0.15
Norway (1)	2004	4.5	22	0.20
Germany (3)	2006	82.5	320	0.25
Switzerland (5)	2006	7.5	22	0.34
Finland (1)	2004	5.3	15	0.35
Sweden (1)	2004	8.9	20	0.45
Switzerland	2015	8	17	0.47
Sweden (4)	2011	9.5	18	0.53
France (2)	2004	60.4	105	0.57
Denmark (1)	2004	5.4	9	0.60
Finland (prediction)	*2015*	*5.2*	*(7 projected)*	*0.74*
**Cities:**				
Lausanne and surroundings, Switzerland	2015	0.8	1	0.8
Fort Myers and surroundings, Lee County, Florida, USA	2010	0.8	1	0.8
Montreal, Canada	2011	2.3	1	2.3
Miami Dade County, Florida, USA	2010	2.5	1	2.5
Phoenix area (Valley of the Sun), Arizona, USA	2010	4.2	1	4.2

(1) Langhelle A, Lossius HM, Silfvast T, Björnsson HM, Lippert FK, Errson A, Soreide E. International EMS: the nordic countries. Resuscitation 2004; 61:9-21.

(2) Adnet F, Lapostolle F. International EMS Systems: France. Resuscitation 2004; 63:7-9.

(3) Roessler M, ZuZan O. EMS systems in Germany. Resuscitation 2006; 68:45-49.

(4) Ek B, Edström P, Toutin A, Svedlund M. Reliability of a Swedish pre-hospital dispatch system in prioritizing patients. Int Emerg Nurs 2013; 21:143–149.

(5) Imbach S. Le Secourisme en Suisse. Service Sanitaire Coordonné, 2008.

Reducing the number of DCs may contribute to improvements in recruiting, training and retaining qualified personnel, and to controlling the high costs of these structures (salaries, computer-aided dispatch systems). A lower number of DCs may, in particular, allow the number of dispatchers covering the same geographical region to be reduced, especially during night-time.

The limit to such concentration is mainly political, as most communities are reluctant to have others managing their EMS. Another limit is represented by languages, as in Switzerland, for example, where there are three linguistic regions: French, Italian and German.

## Conclusion

Streamlining does not mean rationing. The ongoing trend observed in Europe toward reducing the number of DCs has been made possible with the evolution of technology. Streamlining may improve global costs, ease the management of human resources, and improve the quality of continuous education as it is concentrated on fewer dispatchers. As there is today a lack of data on this issue, studies and feedback are needed to allow dispatch science to evolve.
